# Assessing risk for severe domestic violence and related homicides perpetrated by partners and in-laws: adapted danger assessments for women in abusive relationships in India

**DOI:** 10.1186/s12889-024-19364-2

**Published:** 2024-07-13

**Authors:** Bushra Sabri, Jacquelyn C. Campbell, Naseem Ahmad Khan, Mohammad Tahir, Mohd Arif Khan, Mohd Naseem Khan

**Affiliations:** 1 School of Nursing, Johns Hopkins University, 525 North Wolfe Street, Room N530L, Baltimore, MD 21205 USA; 2 Department of Social Work, Aligarh Muslim University, Aligarh, India

**Keywords:** Domestic violence, Homicide, In-laws, Partners, India

## Abstract

Despite domestic violence and related homicides perpetrated by partners and/or in-laws being a significant public health problem in India, there are no reliable and valid instruments to identify and intervene with women in domestic violence relationships. Continued domestic violence can escalate to severe, near-lethal, or lethal violence or homicide. The Danger Assessment (DA) is a risk assessment instrument designed to assess the likelihood of severe, near-lethal, or lethal violence in abusive relationships. However, the DA is not designed to determine the risk of future severe, near-lethal, or lethal violence by in-laws. In-law abuse plays a significant role in domestic violence-related homicides in India and other countries with similar cultural norms. This study addressed this gap by developing the Danger Assessment for in-laws (DA-L) to assess risk from in-laws, alongside the Danger Assessment for Women in India (DA-WI) to assess risk from partners. The study also examined the psychometric properties of the DA-L and DA-WI. Longitudinal data from 150 women in India were used to measure the reliability and validity of the two versions of the DA. The original DA items and additional risk items were examined using relative risk ratios for their relationship with severe violence at three-month follow-ups. Predictive validity was tested with the receiver operating characteristic curve. The study resulted in reliable and valid measures (11 items DA-L and 26-items DA-WI) of risk. The versions of the DA can be useful for practitioners in India and those working with Indian women in the US and other countries. The DAs can be used for identifying women in domestic violence relationships who are at risk for future severe domestic violence and guide the provision of tailored safety plans.

## Introduction

Domestic violence is a significant public health problem in India, with a disproportionally negative impact on women. Although domestic violence can include abuse in marital as well as other family relationships [1], violence against women in marital relationships remains a critical public health issue in India. In a recent 2019–2021 Indian National Family Health Survey report, 32% of ever-married women in India reported domestic violence in marital relationships [2]. In the Indian context, domestic violence is defined as actual or the threat of physical, sexual, economic, and/or psychological harm perpetrated on the woman by her spouse or intimate partner [3] and/or in-laws [4–6] and can result in extreme consequences such as homicides and suicides [7, 8]. Homicides are the most severe outcome of domestic violence, with homicide perpetrated by an intimate partner globally constituting more than a third of female homicides [9].

Various factors contribute to the heightened risk of domestic violence and domestic violence-related homicides in India. These include norms and attitudes that inadvertently support violence against women, male dominance, harmful ideals associated with masculinity, and an emphasis on family honor [10, 11]. Additional risk factors include challenges arising from a partner’s addiction problems, women’s illiteracy or low education levels, lower household income, belonging to a lower caste [12], and cultural beliefs related to gender-based power inequalities, which act as barriers to help-seeking [13]. Other unique cultural risk factors in India include threats to the position of honor, violence related to dowry expectations and demands, and families supporting partner abuse [8]. Thus, there is a critical need to identify women at the greatest risk for experiencing severe or lethal violence in relationships through culturally informed, well-validated danger or risk assessment instruments.

### Need for a culturally responsive measure to assess the risk for future severe domestic violence and related homicides in India

Globally, there is a growing recognition of the need for reliable and valid culturally responsive domestic violence measures [14–17], with a recent review examining the psychometric properties of existing culturally responsive domestic violence measures [15]. Specifically, for the South Asian context, the review identified measures such as the Domestic Violence Questionnaire [18], the Indian Family Violence Coercion Scale [19], the South Asian Violence Screen [20], and the Scale to Measure In-Laws Exploitation and Abuse for South Asian immigrants in the US [14, 15]. However, existing evidence-based domestic violence measures for the South Asian context, including India, are designed to measure the prevalence, types, and dynamics of domestic violence. These measures are *not risk assessment instruments specifically designed to assess risk for future severe*,* near-lethal*,* lethal violence or homicide by partners and in-laws* for women in India.

Women’s diverse cultural backgrounds may shape the understanding, description, and reporting of experiences of domestic violence [15]. Not being culturally informed can lead to inaccurate determination of risk and premature assumptions about women’s risk levels in abusive relationships. Since risk assessments that appear reliable and valid in one culture may not be reliable and valid in other cultures, it is imperative to develop risk assessments for specific cultural groups. Using a “one size fits all approach” to the use of risk assessment instruments such as the Danger Assessment [21] designed to assess risk for future violence or homicide among US-born abused women can lead to inaccurate assessment of risk in Indian setting. This study adds to the literature by developing and testing a danger assessment instrument to predict risk for future severe domestic violence or domestic violence-related homicides among women facing domestic violence in India.

### Rationale for using the danger assessment for Indian cultural setting

Most of the domestic violence risk assessment tools have been developed in Western societies, with more focus on assessing recidivism of partner violence by offenders rather than risk for domestic violence homicide [22]. In a systematic review of literature [23], 18 risk assessment tools were identified that were tested in eight countries (US, Canada, Sweden, Israel, Austria, New Zealand, Spain, and China), with the majority of instruments studied (65%) in the US and Canada. The *Danger Assessment* was found to be one of the most widely studied instruments [23]. The Danger Assessment (DA), focusing on the survivors’ experience and perception [22], is a reliable and valid clinical and research instrument developed in the US to assist women in assessing their danger of being killed or seriously injured by their spouses or partners [21]. The items on the DA determine each woman’s potential risk of being victimized by severe or lethal violence. A weighted scoring system identifies women at different levels of danger: variable danger (< 8), increased danger (8–13), severe danger (14–17), and extreme danger (> 18). Women are then provided with a tailored safety plan based on their level of risk on the DA.

Despite a large number of women being victimized by domestic violence and related homicides in India, prior research has not adapted or tested the DA to identify women at high risk for severe, near-lethal, or lethal domestic violence in India. Also, there is no reliable and valid instrument available to identify women at high risk for experiencing future severe violence by in-laws. In-laws have been found to instigate or support partner violence, directly perpetrate violence, and be complicit in the homicides of women in Indian families [7, 8, 24]. Abuse by in-laws can be attributed to factors such as the need for control in the household, jealousy, fear of losing the son, dowry expectations, and gender norms related to household chores [8]. The patrilocal culture of joint family setups, where a woman co-resides with her husband and his family, can provide opportunities for violence perpetrated by both husbands and in-laws [8, 25, 26]. Husbands and in-laws may reinforce one another’s abuse as they support one another’s entitlement to control women. This entitlement is rooted in traditional patriarchal ideologies that promote the inferiority of women and servitude to the husband and his family members [8, 27]. Thus, there is a need for adaptation of the DA to incorporate unique risks or vulnerabilities of women in the Indian cultural context.

Women experiencing severe domestic violence are at an elevated risk of being killed by their abusers [21, 28]. Therefore, the purpose of this study was to (a) create the DA (Danger Assessment for Abuse by In-Laws-DA-L) to assess risk of future severe violence from in-laws, (b) culturally adapt the DA to assess risk of future severe violence by intimate partners (Danger Assessment for Women in India-DA-WI) within the Indian context, and (c) examine the reliability and validity of the DA-WI and DA-L in assessing future risk for severe domestic violence. This study can be useful for practitioners in conducting culturally informed risk assessments and safety planning with women in India. The findings on DA-WI and DA-L can also be useful for practitioners working with Indian women abroad, as cultural norms related to abuse by partners and in-laws may persist after immigration. These measures can help practitioners identify and address the unique challenges faced by Indian immigrant women who may continue to experience culturally influenced abuse patterns, such as abuse related to dowry demands or a preference for a male child. Additionally, these insights can aid practitioners in supporting abused women in other Asian countries with similar cultural norms.

## Materials and methods

### Identification of culturally relevant risk items and cultural adaptation of the existing version of the danger assessment

The development of an adapted version of the DA for partners and in-laws involved the following steps: (1) The items on the existing version of the DA and DA-I (Danger Assessment for Immigrant Women) were combined and translated into Hindi by a professional translator. The research team members, knowledgeable about the Indian cultural context, evaluated the translated version of the DA for cultural appropriateness. (2) Seventeen in-depth interviews and a focus group (*n* = 10) were conducted with survivors of domestic violence in India. The qualitative data identified risk factors for severe domestic violence and domestic violence-related homicide for inclusion in the DA for the Indian cultural context. For the cultural adaptation of the existing version of the DA, women in India were asked to go through the items on the original DA and items from the DA-I and verbalize their thoughts about each DA item, including cultural appropriateness and understanding. In addition, qualitative data was collected on women’s perceived risk factors for domestic violence and related homicides in Indian families, with the wording of a few items adapted to fit the cultural context. For example, the item on partner’s gun ownership was adapted to replace the word “gun” with knife or other weapon”. Since gun ownership is not common in India, and women are mostly killed using other means (e.g., stabbing, burning), we identified the need to change the risk item on the partner’s gun ownership. (3) Additional risk items were identified by examining characteristics and motives of domestic violence-related homicides and suicide cases (*n* = 100) in India using newspaper reports [6]. (4) Using findings from Steps 2 and 3, two different versions of the DA were developed to assess the risk for severe violence perpetrated by partners and in-laws and to examine the reliability and validity of the two versions of the DA for the Indian cultural context.

### Longitudinal data collection to assess the reliability and validity of the danger assessment

Using a longitudinal design, quantitative survey data were collected from 150 abused women in rural areas of Aligarh district in North India from August 21st, 2021, to March 21st, 2022, at baseline and three months follow-up. Women were recruited with support from staff at the community health centers providing prenatal and post-natal healthcare services, Block and district health officials, community health workers, known as Accredited Social Health Activists (ASHAs), as well as with support from local Pradhan (the elected head of the village [29] from each village). Healthcare services can serve as an ideal platform for domestic violence-related risk assessment and intervention for marginalized and underserved groups of women in India, particularly those residing in rural areas. Women in rural settings in India often lack awareness of and access to dedicated domestic violence services. Additionally, the stigma surrounding domestic violence as a private family matter can further hinder their disclosure or help-seeking behaviors. Healthcare settings can provide a trusted and safe environment for women to disclose domestic violence, receive immediate medical attention if needed, and connect with other community resources [30]. Despite the majority of the population in India residing in rural areas (65% in 2021 [31]), services for domestic violence remain predominantly concentrated in urban regions. Therefore, the DAs for partners and in-laws were initially tested among rural women.

Women were invited to participate in community-based health clinics, health camps, and community events in rural sites using on-site trained Research Assistants (RAs). ASHAs also shared the study information with the women they worked with. ASHAs, residents of the area where they provide health services, had a good rapport with women through healthcare home visits. They were key facilitators for data collection and retaining women for the follow-up survey. Therefore, all participants who completed baseline were retained at three months follow-up. Trained RAs, with assistance from ASHAs when necessary, screened interested women for eligibility. To be eligible for participation, women had to be over 18 years of age, had to have experienced intimate partner and/or in-law abuse within the past year, and had to be residing in rural or tribal areas in India. Women were screened for eligibility using an electronic screener that included questions adapted from the Abuse Assessment Screen [32]. RAs assisted women in completing the computerized survey at a private, safe, and convenient location. The computerized survey (approximately 60 min) was administered verbally by RAs to those women who could not read or clearly understand the survey questions. Women who were able to read completed the survey on their own. The survey questions focused on demographics and relationship characteristics, the original DA items, additional risk items identified in prior qualitative work [8], and the type, frequency, and severity of abuse by partners and in-laws. All women were compensated 100 Indian rupees (about 2 USD) for their time in completing the surveys.

### Ethics statement

The study was approved by the Johns Hopkins School of Medicine Institutional Review Board and the Research Ethics Committee of Aligarh Muslim University, India, to ensure compliance with ethical standards and protect study participants. RAs received extensive training on study implementation, ethical considerations in conducting research with survivors of domestic violence, including informed consent procedures, and ensuring the protection of privacy and confidentiality of women and their data. All participants provided oral consent before participation in the survey, which was conducted one-on-one in person in a safe and private location agreed upon by both parties. Participants were identified by study code numbers, and all identifying information was omitted from the study database. All participants were provided with or referred to support services as needed.

## Measures

### Outcomes

#### Severe intimate partner violence and in-law abuse

Severe intimate partner violence and in-law abuse were assessed at baseline and three months follow-up using the Revised Conflict Tactic Scale (CTS2) [33] for the partner and adapted version for the in-laws [34]. For in-law abuse, the questions were asked referring to in-laws as perpetrators. The CTS2, a reliable and valid self-report instrument, is one of the most commonly used measures of intimate partner violence. Intimate partner violence and in-law abuse were considered severe if women answered “yes” to severe domestic violence questions from the physical violence section of the CTS2: (1) choked her, strangled her or cut off her breathing, and (2) attacked her with a knife or any weapon.

### Risk factors

#### Danger assessment (DA)

The DA, a reliable and valid self-report instrument administered at baseline, is an instrument designed to assess women’s risk for intimate partner homicide and empower women to make decisions about their safety. Risk for future violence was assessed using 22 yes/no items selected from both the original DA [21] and its adapted version for immigrant women in the US [28]. Example DA items included: “Has the physical violence increased in severity or frequency over the past year?” b) Have you ever been beaten by him while you were pregnant?

#### Additional risk items

Using findings from the qualitative work with women in India and an analysis of domestic violence homicides and suicides in India from the newspaper reports (described above), an additional 32 risk items were included in the baseline survey (Table 1). These included dichotomous (yes/no) items such as in-laws making false allegations, threatening to harm or kill based on family honor, abusing for dowry, partner having anger problems, partner, or in-laws’ use of black magic, spreading rumors about her, abusing her for not giving birth to a female child and others. Example items include: “Has he ever scared you with his behavior or body language (e.g., making you watch movies or telling horror stories where the man killed his wife who did not listen to him)”? “Does your partner keep necessities from you, such as food or personal care items”? Have you ever been mistreated for not being able to get pregnant?” “Does your in-laws support your partner’s abuse?”

**Table 1:** Additional risk items

1.	Partner witnessed violence in his home while growing up
2.	He experienced mistreatment by his parents and other family members in childhood.
3.	He/in-laws used black magic or other cultural practices to harm her.
4.	He is unfaithful/has other sexual/intimate partner relationships.
5.	He has multiple wives.
6.	He has serious anger issues or problems controlling his anger/temper.
7.	He has mental health issues.
8.	He has children outside the marriage.
9.	He abuses her for dressing or behaving like a modern woman.
10.	He shows sudden changes in mood/behavior.
11.	She always needs permission from her in-laws to do things.
12.	She is abused for giving birth to a female child.
13.	She always needs permission from her partner to do anything.
14.	She is scared of people’s reactions to her being abused.
15.	He/in-laws threatened to harm/kill her for not having a son.
16.	He/in-laws abused her for dowry.
17.	He/in-laws threatened to harm/kill for dowry.
18.	She feels pressured to stay in the abusive relationship because of religion/culture.
19.	He keeps necessities from her (e.g., food)
20.	He scares her with his behavior/body language.
21.	He/his family spreads rumors about her (e.g., crazy)
22.	He/his family makes false accusations.
23.	Her own family/community rejects her because of his/her in-laws’ false accusations.
24.	The husband/partner isolates her from family/friends.
25.	In-laws support partner’s abuse
26.	He/in-laws locked her/held her against her will.
27.	She is abused for not being able to get pregnant.
28.	He/in-laws did not care for healthcare needs.
29.	He/in-laws threatened to harm/kill for family honor.
30.	He/her in-laws threatened to harm/kill her for leaving.
31.	She feels the need to hide her husband’s abuse from family/community/friends.
32.	Her own family/community members do not support her.

#### Self-perceived risk

At baseline, women were asked to rate the likelihood on a scale of zero to ten, with zero meaning no chance and ten meaning sure it will happen that their husband/partner and/or in-laws will physically hurt her in the near future.

### Socio-demographic variables

At baseline, women were asked single-item questions on socio-demographic characteristics such as age, marital status, education, number of children, and employment status.

## Data analysis

Data were analyzed using descriptive statistics. Relative risk ratios (RRRs) were used to examine the bivariate relationships between the items on the Danger Assessment (DA) and additional risk items and the outcome of severe intimate partner violence and in-law abuse at three months follow-up. RRRs were used in estimating future risk for severe violence based on an affirmative response to a particular risk factor. An RRR of 1 indicates no risk, an RRR below 1 indicates a decreased risk, and an RRR above 1 indicates an increased risk for severe intimate partner violence or in-law abuse. The RRRs are also used to identify the relative strength of each risk item and how heavily a particular risk item needs to be weighted in the risk assessment [28].

The unweighted and weighted versions of the DA for Women in India (DA-WI) to assess risk for partner violence were created. The scoring involved counting the “yes” responses to each item, with a higher number of “yes” responses indicating the presence of more risk factors. For weighted versions, weights were assigned to risk items based on the RRRs by using the formula developed in prior work on adaptations of the DA [28, 35]. Items with an RRR below 1.33 were given a weight of 0. Items with an RRR of 1.33 to 1.79 were assigned a weight of (1) Items with RRR ranging from 1.80 to 2.79 were assigned a weight of (2) Those with an RRR of 2.80 to 3.79 were given a weight of 3, and those with an RRR of 3.80 and higher were given a weight of 4. Therefore, only items that were significant with an RRR of 1.33 were retained in the adapted DAs for women in India for partner abuse (DA-WI) and in-law abuse (DA-L). In DA-WI, the weighting of the item on strangulation was increased from 2 to 3 due to it being a significant risk factor for femicide in multiple studies [36] and it being also significantly associated with severe in-law abuse (among two participants) in this sample.

Logistic regressions were then conducted on the baseline unweighted and weighted versions of the DA-WI and DA-L to predict severe intimate partner violence and in-law abuse three months later. We adjusted for covariates significantly related to the outcomes of severe intimate partner violence and in-law abuse. We assessed the concurrent validity of the DA-WI by examining correlations between the original DA score and the DA-WI, as well as women’s self-reported risk and the DA-WI. The concurrent validity of the DA-L was examined using correlations between the DA-L and women’s self-reported risk for experiencing future violence. Internal consistency reliability of the DA-WI and DA-L was tested using Cronbach’s alpha. All analyses were conducted using SPSS version 27.

The Receiver Operating Characteristic (ROC) curves were used to assess if the DA-WI and DA-L were useful in correctly identifying women at risk for severe intimate partner violence and in-law abuse. For this, the continuous DA-WI and DA-L scores were used with the binary outcomes of severe violence at three months follow-up. The ROC curves present sensitivity and 1-specificity of a measure for every possible range of cut-off values that could be a potential threshold for designating a participant who is at high risk [21]. Sensitivity refers to the percentage of women who are at high risk for experiencing severe intimate partner violence or in-law abuse in the future and correctly identified by the DA-WI and DA-L. Specificity refers to the percentage of women not at high risk and correctly excluded by the DA-WI and DA-L assessments. The ROC curve plots sensitivity as a function of the false-negative rate, thereby considering sensitivity and the converse (false-positive rate) as well as specificity and the converse (false-negative rate) [28, 35].

The approach involves examining the proportion of the graph that lies under the plotted ROC curve (area under the curve (AUC)). The AUC is the probability that a randomly chosen member of group 0 will produce a score lower than the score of a randomly drawn member of group 1. The values of AUC range from 0 to 1.0, with 0 indicating that no cases were predicted accurately and 1 suggesting that every case is predicted accurately. The AUC equivalent of effect size classifications is calculated as follows: an AUC of 0.556 corresponds to a small effect (*d* = 0.2), an AUC of 0.639 corresponds to a medium effect (*d* = 0.5), and an AUC of 0.714 corresponds to a large effect (*d* = 0.8) [28, 37]. The AUC and Youden’s index were used to aid the selection of the most suitable cut-off values for the DA-WI and the DA-L.

## Results

### Sample characteristics

Table 2 presents participant characteristics.

**Table 2:** Participant characteristics (*N* = 150)

Age, years (Mean) (SD)	25.03 (4.13)
*Husband’s age* (Mean, SD)	28.79 (4.90)
*Age at marriage* (Mean, SD)	18.56 (2.57)
*Education (*%, N)	
Did not attend school or did not complete primary school	45.3 (68)
Completed primary school (1-5th grade)	16.0 (24)
Completed secondary school (6-9th grade)	21.3 (32)
Studied until 10th or 12th grade	14.7 (22)
Completed Bachelor’s degree or higher	2.6 (4)
*Frequency of Financial Stress* (%, N)	
Less than monthly	38.7 (58)
Monthly or more frequently	47.4 (71)
Did not know	14.0 (21)
*Relationship Status* (%, N)	
Married	94.0 (141)
Widowed/Divorced and Remarried	3.4 (5)
Separated or in the process of separation	0.14 (2)
Widowed not remarried	1.3 (2)
*Co-residing with In-Laws* (%, N)	76.7 (115)
*Currently employed* (%, N)	9.3 (14)
*Husband’s Employment (Past 12 months)* (%, N)	
Yes, currently employed	45.3 (68)
Yes, but currently, unemployment	30.7 (46)
Unemployed in the past 12 months	24.0 (36)
*Number of children in the home* (%, N)	
0	8 (12)
1 2	30 (45) 28 (42)
3	22.7 (34)
4+	11.3 (17)
*Currently Pregnant (*%, N)	28.7 (43)

The average age of women was 25.02 (*SD* = 4.13), with average age of marriage being 18 (*SD* = 2.57). Most women (89.3%; *n* = 134) were educated till high school (10th grade) or less, with a large proportion being those who did not attend or complete primary school (45.3%, *n* = 68). Only four women had a Bachelor or Master degree (2.6%, *n* = 4). The majority of women were married (94%, *n* = 141), with most of them co-residing with the in-laws (76.7%, *n* = 115) and having two or more children (62%, *n* = 93). Employment was not common. Only 9.3% of women in the sample were employed (*n* = 14) (Table 1).

### Relationships of baseline risk assessment items and women’s experiences of severe intimate partner violence and in-law abuse at three months

The significant bivariate associations of the original DA items and the additional risk items with any severe intimate partner violence at three months are presented in Table 3.

**Table 3:** Relative risk ratios for DA-WI items (*N* = 150)

		Relative risk ratios (95% CI)	
	Risk Assessment Items	Severe IPV (*N* = 58; 39%)	Weight
	**Original Danger Assessment Items**		
1	Increase in severity/frequency of physical violence	1.90 (1.00-3.61)	2
2	He threatened to kill	1.76 (1.19–2.58)	1
3	He is constantly/violently jealous	3.32 (1.63–6.76)	3
4	She left him in the past year	2.32 (1.56–3.43)	2
5	Used/threatened with a lethal weapon	2.64 (2.15–3.24)	2
6	Lied about behavior/avoided being arrested	3.52 (1.80–6.85)	3
7	Choking/Strangulation	2.05 (1.30–3.22)	3
8	Partner uses illegal drugs	1.86 (1.21–2.85)	2
9	He is an alcoholic/problem drinker	1.87 (1.13–3.09)	2
10	She was beaten while pregnant	5.60 (1.46–21.4)	4
11	Partner is capable of killing her	2.04 (1.38-3.00)	2
12	She threatened/tried suicide	1.67 (1.13–2.46)	1
	**Additional Risk Items**		
13	He keeps necessities from her (e.g., food)	10.32 (1.50–70.8)	4
14	He scares her with his behavior/body language	2.23 (1.44–3.44)	2
15	He/his family spreads rumors about her (e.g., crazy)	1.70 (1.14–2.52)	1
16	He/his family makes false accusations	2.37 (1.54–3.62)	2
17	Rejection by her own family/community because of his/in-laws’ false accusations (e.g., sleeping around)	2.41 (1.29–4.47)	2
18	Husband isolates her from family/friends	2.69 (1.53–4.75)	2
19	In-laws support partner’s abuse	9.83 (2.51–38.4)	4
20	Abuse for not being able to get pregnant	5.58 (1.86–16.7)	4
21	He/in-laws did not care for healthcare needs	4.12 (1.60–10.5)	4
22	He/in-laws threatened to harm/kill for family honor	2.83 (1.32–6.06)	3
23	He/her in-laws threatened to harm/kill her for leaving	2.11 (1.35–3.29)	2
24	She feels the need to hide her husband’s abuse from family/community/friends	2.47 (1.23–4.97)	2
25	Family/community members lack of support	2.65 (1.16–6.04)	2
26	Her parents/siblings support her abuse	2.81 (1.50–5.24)	3

The Relative Risk Ratios indicate that 26 items are to be retained for the DA-WI (12 out of 21 items from the original DA and 14 out of 32 additional risk items). These 12 original DA items predictive of future severe intimate partner violence included: past year increase in frequency and severity of violence, partner’s threats to kill, constant and violent jealous behavior, use or threats of use of a lethal weapon, choking or strangulation, lying about abusive behavior or avoiding arrest, problem with alcohol and drugs, physical abuse during pregnancy, her history of leaving him after living together, her suicide ideation/attempts and her perception of partner capable of killing her. Fourteen new items were identified as predictive of severe intimate partner violence at three months with the highest weight (i.e., 4) assigned to items on partner keeping necessities from her (e.g., food) (RRR = 10.32), in-laws supporting partner’s abuse (RRR = 9.83), her being abused for not being able to get pregnant (RRR = 5.58) and husband or in-laws not caring for health care needs (RRR = 4.12). Other significant items were threats to harm or kill her for family honor (RRR = 2.83), for leaving (RRR = 2.11), or for her own parents and siblings supporting her abuse by her partner and in-laws (RRR = 2.81) (Table 3).

Table 4 presents significant bivariate associations of the DA items and additional risk items with severe in-law abuse at three months.

**Table 4:** Relative risk ratios for DA-L items (*N* = 150)

		Relative Risk Ratios (95% CI)	
	Risk Assessment Items	Severe In-Law Abuse (20%, *n* = 30)	Weight
	**Danger Assessment Items**		
1.	Increase in severity/frequency of physical violence	4.26 (1.07-17.0)	4
2.	Threats to kill	1.90 (1.01–3.57)	1
3.	Constant/violent jealousy	4.10 (1.31–12.8)	4
4.	Forced sex	2.67 (1.12–6.36)	2
5.	Choking/Strangulation	2.73 (1.26–5.92)	4
6.	Partner uses illegal drugs	2.46 (1.21–5.01)	2
	**Additional Risk Items**		
7.	He/her family makes false accusations	3.13 (1.54–6.38)	3
8.	Husband isolates her from family/friends	2.52 (1.09–5.79)	2
9.	He/in-laws locked her/held her against her will	2.36 (1.22–4.56)	2
10.	Abuse for not being able to get pregnant	4.26 (1.07-17.0)	4
11.	Abuse for dowry	2.40 (0.94–2.08)	

Eleven items were retained for DA-L that were predictive of severe in-law abuse. These included six partner’s behavior items from the original DA with the highest weight of 4 assigned to increase in severity of frequency of violence, jealous behavior, and choking or strangulation. Additional risk items for future severe in-law abuse included abuse for not being able to get pregnant (RRR = 4.26), spreading of rumors about her or making false accusations (e.g., she is sleeping around) (RRR = 3.13), isolating her from family or friends (RRR = 2.52) and locking her up against her will (RRR = 2.36). An item on her abuse due to dowry was included in the initial analysis because of the strong association between dowry demands by in-laws and risk for homicide in the Indian cultural context [8], even though we found a statistically non-significant RRR for both partner and in-law abuse. In further analysis, the dowry item did not significantly impact the predictive validity of the DA-L. Therefore, the findings for DA-L are presented, excluding the dowry item.

### Reliability and validity of the danger assessment of women in India (DA-WI)

Table 5 below shows that both unweighted and weighted versions of the DA-WI are significant predictors of future severe intimate partner violence.

**Table 5:** Logistic regression findings: DA-WI for severe intimate partner violence

			Risk for severe IPV	
Predictors	Mean (SD)	Scores	OR (95% CI)	*p*-value
Unweighted DA-WI	14.26 (6.35)	00 to 26	1.244 (1.14 to 1.35)	0.000
Weighted DA-WI	38.29 (16.0)	00 to 64	1.104 (1.06 to 1.15)	0.000

Each additional risk factor on the unweighted DA-WI was associated with a 1.244 increase in the odds of severe intimate partner violence. For each additional risk factor on the weighted DA-WI, there was a 1.104 increase in the odds of severe intimate partner violence. In the model adjusting for covariates (i.e., education, age, and age of marriage), the weighted DA continued to be a significant predictor of severe intimate partner violence (*OR* = 1.102, *p* = .000). Table 6 shows that the ROC AUC for the DA-WI weighted score was 0.803, corresponding to a large effect size [37].

**Table 6:** Receiver operating characteristic area under the curve (AUC) (95% CI) -DA-WI

Severe intimate partner violence	*N*	Unadjusted AUC (CI)	*p*-value	Adjusted AUC (CI)	Adjusted *p*-value
Unweighted DA-WI	150	0.679 (0.57 to 0.78)	0.002	0.804 (0.73 to 0.87)	0.000
Weighted DA-WI	150	0.803 (0.73 to 0.87)	0.000	**0.815 (0.74 to 0.88)**	0.000
Perception of future risk	143	0.737 (0.65 to 0.81)	0.000	0.759 (0.68 to 0.83)	0.000
Original DA	150	0.741 (0.66 to 0.82)	0.000	0.766 (0.69 to 0.84)	0.000

The AUC of the DA-WI was also larger than the AUC of women’s self-perceptions of future risk (AUC = 0.737) and the original DA (AUC = 0.741). After adjusting for the covariates, the AUC for the weighted DA-WI remained higher than others and increased to 0.815. Significant correlations of the weighted DA-WI with the original DA score (*r* = .86; *p* = .000) and women’s self-reported risk (*r* = .77; *p* = .000) established concurrent validity of the DA-WI. The internal consistency reliability of the 26-item DA-WI was established by Cronbach’s alpha of 0.91.

### Reliability and validity of danger assessment for in-law abuse (DA-L)

Table 7 below shows that both unweighted and weighted versions of DA-L are significant predictors of future severe in-law abuse.

**Table 7:** Logistic regression findings: risk assessment for abuse by in-laws (DA-L) for severe in-law abuse

			Risk for severe in-law abuse	
Predictors	Mean (SD)	Range of Scores	OR (95% CI)	*p*-value
Unweighted DA-L	14.26 (6.35)	00 to 26	1.540 (1.23 to 1.92)	0.000
Weighted DA-L	13.20 (6.79)	00 to 28	1.192 (1.09 to 1.30)	0.000

Each additional risk factor on the unweighted DA-L was associated with a 1.540 increase in the odds of severe abuse by in-laws. For each additional risk factor on the weighted DA-L, there was a 1.192 increase in the odds of severe in-laws’ abuse. In the model adjusting for covariates (i.e., age and number of children), the weighted DA-L continued to be a significant predictor of severe in-law abuse (*OR* = 1.182, *p* = .000). Table 8 shows that the ROC AUC for the DA-L weighted score was 0.76, corresponding to a large effect size [33].

**Table 8:** Receiver operating characteristic area under the curve (95% CI) -DA-L

Severe in-law abuse	*N*	Unadjusted AUC (CI)	*p*-value	Adjusted AUC (CI)	Adjusted *p*-value
Unweighted DA-L	150	0.743 (0.64 to 0.84)	0.000	0.771 (0.68 to 0.86)	0.000
Weighted DA-L	150	0.764 (0.67 to 0.85)	0.000	0.786 (0.69 to 0.87)	0.000
Perception of future risk	143	0.683 (0.58 to 0.78)	0.002	0.751 (0.66 to 0.83)	0.000

The AUC of the DA-L was also larger than the AUC of women’s perceived risk for experiencing future violence (AUC = 0.683). The concurrent validity of weighted DA-L was demonstrated by a significant correlation with women’s self-reported risk (*r* = .76; *p* = .000). Further, the Cronbach’s alpha of the 10-item DA-L excluding the dowry item being 0.78 and 11-item DA-L including the dowry item being 0.79 established the internal consistency reliability of the measure assessing risk for future severe in-law abuse.

### Exploring optimal cut-off for the DA-WI and DA-L

The optimal cut-off values for differentiating high-risk women from those at no or low risk were examined for both DA-WI and DA-L (Tables 9 and 10).

**Table 9:** Possible cut-off values for DA-WI

Possible cut-off scores	Sensitivity (True positives) (%)	False positives (1-specificity)	Specificity (True negatives)	Positive predictive value	AUC	Youden’s statistic
**37.50**	**91.4%**	**42%**	**58%**	**68.4%**	**0.752 (0.66 to 0.83)**	**0.49**
33.50	93.1%	55.4%	45%	62.6%	0.726 (0.63 to 0.81)	0.38
35.50	91.4	46%	54%	66.5%	0.746 (0.66 to 0.83)	0.45

**Table 10:** Possible cut off values for DA-L

Possible cut-off scores	Sensitivity (True positives) (%)	False positives (1-specificity)	Specificity	Positive predictive value	AUC	Youden’s statistic
**15.5**	**77%**	**35.8%**	**64.2%**	**68.7%**	**0.737 (0.63 to 0.83)**	**0.41**
14.5	77%	41.7%	58.3%	56.9%	0.737 (0.63 to 0.83)	0.35
16.5	73.3%	29.2%	71%	71.5%	0.737 (0.63 to 0.83)	0.44

A value with the highest Youden’s index and maximum area under the curve (AUC) was chosen for the cut-off. Table 8 shows that for the DA-WI, the cut-off value of 37.50 has a very high sensitivity (91.4%), reasonable specificity (58%), a positive predictive value of 68.4%, and the highest Youden’s index (i.e., 0.49). With this value, approximately 91% of the cases will be correctly predicted by the DA-WI if the scores are above or equal to 37.50, and 68.4% of women who meet the criteria of being at high risk will actually be at high risk for experiencing severe violence.

For DA-L, the cut-off value chosen is 15.5 due to its high sensitivity of 77%, reasonable specificity of 64.2%, positive predictive value of approximately 69%, and AUC of 0.737. Although the Youden index (combination of sensitivity and specificity) for this cut-off value is below the highest value of 0.44 (i.e., 0.41), we selected the cut-off of 15.5 because of sensitivity being important than specificity [38]. Table 9 below shows possible cut-off values for DA-L.

## Discussion

Our study is the first to adapt and assess the psychometric properties of two versions of the Danger Assessment (DA): The Danger Assessment for Women in India (DA-WI) for partner-related risk and the Danger Assessment for risk from in-laws (DA-L), designed to evaluate women’s risk of future severe violence from partners and in-laws in India. In our study, the 26-item DA-WI (12 out of 22 items from the original DA and 14 additional risk items) appeared to provide significantly greater accuracy in predicting risk for near-severe intimate partner violence for women than the original DA. Additionally, it was more accurate than women’s self-perception of their future risk of intimate partner violence. Similarly, the 11-item DA-L (6 items from the original DA and 5 additional risk items) appeared to be a more accurate predictor of severe in-law abuse than women’s self-perception of future risk of violence by in-laws. While 12 partner-related risk factors from the original DA were included in the DA-WI (e.g., increase in severity or frequency of violence, alcohol use) [21], our findings led to the exclusion of 9 risk factor items. The risk factors that were excluded included the partner’s control of her daily activities, the partner’s suicide threats, his ownership of a weapon, his threats to harm children, his unemployment, and a history of forced sex perpetration. Women having a child that was not his, being ashamed of the things their partner did to them, or hiding the truth from others were additional factors that were excluded from the original DA.

In another study, partner’s sexually abusive behaviors were related to an increased likelihood of women experiencing severe intimate partner violence and injuries [24]. However, in this study, the perpetration of forced sex was not found to be significantly related to the risk for future severe intimate partner violence. In Indian marriages, marital rape is not legally recognized as a crime, and laws are discriminatory against women who have been raped by their husbands [39]. Cultural norms that expect women to be submissive to their partners and discourage labeling non-consensual sex in marital relationships as ‘forced’ can prevent women from recognizing and reporting marital rape [40, 41]. The findings on non-significance of control could be attributed to the widespread prevalence and normalization of controlling behaviors of men in marital relationships. Such behaviors may not necessarily be classified as abusive in the Indian cultural context unless accompanied by other forms of abuse. However, their husband isolating them from family and friends was found to be significantly associated with a risk for future severe violence. Also, contrary to the findings of the significance of unemployment as a risk factor for severe violence in the US [21], in this study, partner unemployment was not found to be a significant risk factor for inclusion in the DA-WI. Partner’s unemployment and women’s financial dependence can be an added stress in families that can result in day-to-day abuse [8] but may not be significantly associated with future risk for severe domestic violence.

While threats to kill were a significant factor, the threat of a partner’s self-harm or suicide was not found to be significant in our study. This contrasts with research findings in the US, where partners’ suicide threats were significantly associated with women’s risk for victimization by severe or lethal violence [21]. However, our study found that women who exhibited suicidal behavior were more likely to experience future severe intimate partner violence. This suggests that women’s threats or attempts at suicide may be a reaction to their ongoing experiences of severe violence. Prior studies in India have also identified domestic violence as a significant risk factor for women’s suicide attempts, with those in abusive relationships being 2.44–2.60 times more likely to attempt suicide than other women [42].

Women whose partners owned a lethal weapon were also not more likely to experience future severe intimate partner violence than other women. This finding contrasts with research in the US, where gun ownership by abusive partners is a significant factor in IPV homicides and greatly increases the risk of severe or lethal violence against women [21]. It appears that in Indian households, partners and in-laws’ abusive behaviors pose a greater risk than ownership of a weapon. Firearm ownership is not common in Indian households. Domestic violence homicides have been perpetrated using kitchen knives, burning, or strangulation methods, which are commonly available means in homes. In prior work on domestic violence homicides in India, most women were killed by burning or strangulation methods [7]. Our findings on choking or strangulation are in line with the prior work in the US [36]. Women with histories of severe violence in the form of choking or strangulation by a partner were also more likely to be revictimized in the form of severe intimate partner violence as well as severe in-law abuse.

Abuse during pregnancy was also a significant risk item in the DA-WI which was retained from the original DA. This is in line with research in the US [43], where abuse during pregnancy was found to be a significant predictor of severe intimate partner violence. Some unique risk factors were identified for the Indian cultural context in this research. In-laws’ behaviors, such as false accusations, supporting her abuse by the partner, threatening to harm her for family honor, and her own parental family and community’s lack of support, appeared as additional risk factors for women’s exposure to severe intimate partner violence. In a collectivist culture such as India, the emphasis on family reputation, honor, prescribed gender roles, and the significant role of in-laws and parental family in women’s marital relationships can impact women’s and partner’s behaviors and women’s experiences in marital relationships. In many instances, family and community can justify violence against women who deviate from the prescribed norms and bring shame to the family. In prior research in India, patriarchal cultural norms that are related to rigid gender role expectations and normalization of violence against women under certain circumstances have been identified as factors that place women at risk for repeat violence, severe violence, or homicides perpetrated by partner and/or in-laws [8].

Six partner behavior items from the original DA were also found to be significantly related to women’s experience of future severe in-law abuse. In our study, women who experienced husband/partner’s behaviors (items on the original DA), such as an increase in severity or frequency of violence, threats to kill, choking, and misuse of drugs, were also more likely to experience future severe abuse by in-laws. Husbands or partners can become more abusive due to the significant influence of in-laws in Indian families. Co-residing with abusive in-laws can have a negative impact on marital relationships. In prior qualitative work in India [8], women identified a partner’s engagement in violence at the instigation of his family (her in-laws) as a significant risk factor for severe or lethal violence by a partner. The findings highlighted the prominent role of in-laws in the cycle of abuse, with abuse mostly related to gender-role expectations and dowry demands and husbands not believing women’s disclosure of abuse by in-laws [8]. Although women who experienced forced sex were not significantly more likely to experience future severe violence by their husbands/partners than those who did not, they were more likely to experience severe abuse by their in-laws. This could be due to the men’s need for forced sex in marital relationships, considered an indicator of women challenging traditional gender norms. Sex in marriage is considered a husband’s right, and women’s resistance to sex can be perceived as women’s deviation from their duties as a wife and can lead to women’s abuse by in-laws.

This study identified additional risk factors for severe in-law abuse that were specific to in-laws’ behaviors, such as false accusations, locking her against her will, and abusing her for not being able to get pregnant. While her being abused for not being able to get pregnant was not a significant predictor for severe intimate partner violence, it emerged as a significant predictor for future severe in-law abuse. The social norms in India put a lot of emphasis on childbearing, where women, at times, can be blamed for their inability to conceive regardless of their husband’s infertility problem. Marital couples often face significant pressure from their families to have a child who would carry forward the family’s name. In some cases, the in-laws may have more of a desire to have a grandchild than the husband, which may explain it being significantly related to severe in-law abuse and not severe intimate partner violence. Dowry was not identified as a significant predictor of future severe intimate partner violence or in-law abuse. However, we added this as an item based on our prior work on domestic violence homicides in India and women’s perception of dowry being a risk in a qualitative study. It appears that women who experience an increase in frequency and severity of intimate partner violence, as well as other forms of partner and in-law abuse due to dowry and other reasons, could be more at risk for future severe intimate partner violence and in-law abuse than dowry demands without other forms of abuse.

An important strength of the study is the use of longitudinal data to create a DA to predict the risk for future severe violence among women in abusive relationships in India. The study’s limitations include using a small sample size that was determined based on budget and feasibility considerations. Our preliminary findings would need to be replicated with a larger sample size. However, some of our quantitative findings on risk factors are in line with prior quantitative research conducted outside India (e.g., choking and abuse during pregnancy) [36, 43]. Other findings that supported the inclusion of unique risk items in the Indian versions of the DA were based on qualitative research conducted with women in India. The other limitation is using a rural sample of women in North India. However, with 65% of the total population of India residing in rural areas [31] and the cultural adaptation of the DA based on data from both rural and urban women across India, the DA can be generalizable for the broader population in India. Despite the geographical and cultural diversity across India, a review of the literature of studies conducted in diverse regions in India identified commonalities in risk factors for domestic violence (e.g., gender inequitable norms and lack of social support [10]). Further, the items selected in the DA are characteristics of common abusive behaviors and red flags in relationships that can place women at risk for future severe violence. Thus, the study contributes to the literature on culturally informed risk assessments.

The exclusion of certain items from the original DA and the addition of new risk items for women in India highlight the importance of tailoring the risk assessment instruments for different cultural contexts. This study developed instruments to assess risk from both husband and in-laws because many women in India co-reside with their in-laws, and in-laws play a significant role in marital relationships. The DA-L can be administered by healthcare or social service practitioners in cases where women are co-residing with the in-laws or in-laws have a significant influence on their lives. Practitioners can use an empowerment approach to administer the DA-WI and DA-L, educate women about their risks and how to recognize signs of increased risk, and work with them to develop a safety plan. Women can be assisted in making informed decisions about their safety, with healthcare or social service practitioners respecting their choices and the need to keep the family together. Research shows that abused women may underestimate their risk for future revictimization [28]. Although women’s perceptions of risk can be important, the use of DA-WI and DA-L can provide a more realistic assessment of their risk. Risk-informed safety planning and linking women to support services protect women from future harm. Such assessments are critically needed by organizations providing healthcare services to women in India. Further, prevention and intervention efforts are needed to address gender norms and educate the community about the impact of abuse on women’s health and safety.

### Implications for research and practice

Future research can evaluate the effectiveness of administering the versions of the DA and providing tailored safety planning to women based on their level of risk or response to the risk items on the DA in diverse regions and settings in India. The use of the DA and tailored safety planning has the potential to intervene early with high-risk women in India and prevent severe violence or related homicides in Indian families. Further, with increasing emphasis on culturally informed assessments and interventions, the DA-WI and DA-L can also be useful for social service and healthcare practitioners working with Indian women in the US and other countries outside India. Therefore, future research is needed to validate DA-WI and DA-L among Indian women abroad. Studies among South Asian survivors of intimate partner violence in the US have reported increased abuse from in-laws [27, 44], with research also developing and testing measures for in-law abuse among South Asian families in the US [14]. Research has also examined common and culturally specific risk factors for future severe violence and homicide among immigrants in the US, with in-law abuse as one of the risk factors [45]. Practitioners can use culturally informed DAs to identify women at high risk for future severe violence or homicide perpetrated by partners and/or in-laws.

Before administering the DA-WI and DA-L, it would be important for practitioners to emphasize privacy and confidentiality and provide women with a safe space to disclose without fear of retaliation from the abusive partner or his family. Using a trauma-informed and empowerment approach, practitioners could then provide information and help women make informed decisions about their safety. Practitioners can educate women about the risk factors in their situations and provide safety planning based on their specific risk factors. For women at high risk of future severe violence or homicide, practitioners can inform women about risk factors for homicide, how to recognize signs of increased risk, and work with them to develop an emergency plan [28]. Given that healthcare providers are often the first point of care for many women in India, it is crucial to integrate routine screening and support for women in abusive relationships in healthcare settings. This is especially needed in rural areas, where women face limited to no access to DV resources due to factors such as lack of knowledge, financial dependency, and transportation barriers [30]. Healthcare providers can connect women to resources such as DV helplines, temporary shelters, legal aid services, non-government organizations, local women’s groups, and Mahila Police Volunteers, who act as a link between the police and the community to assist women in distress [46]. Additionally, healthcare providers can offer immediate medical care, emotional support, and safety planning to women in abusive relationships. Collaboration between healthcare and social service providers can ensure timely responses to women facing violence and those at risk for repeat violence or homicide.

## Conclusion

This study identified unique risk factors for severe and near-lethal domestic violence for inclusion in risk assessments among women in abusive relationships in India. In collectivist cultures where women are co-residing with in-laws, in-laws play a significant role in women’s experiences of abuse as well as their being victimized by domestic violence homicides. However, there is no danger assessment instrument available for in-laws. This study created a danger assessment instrument for in-laws as well as culturally tailored the existing danger assessment instrument focusing on intimate partners to incorporate unique risk factors for lethal violence perpetrated by partners.

## Data Availability

Data is provided within the manuscript.

## Abbreviations

IPV: Intimate partner violence
DV: Domestic violence
DA: Danger assessment
DA-WI: Danger assessment for assessing risk for partner abuse among women in India
DA-L: Danger assessment for in-law abuse
DA-I: Danger assessment for immigrant women
CTS-2: Revised Conflicts and Tactics Scale
IRB: Institutional Review Board
AUC: Area under the Curve
CI: Confidence Interval
RRR: Relative Risk Ratio
OR: Odd Ratio
N: Sample Size
SD: Standard Deviation
US: United States
RA: Research Assistant
ROC: Receiver Operating Characteristic
ASHA: Accredited Social Health Activist
